# Paternal Exposure to Endocrine Disrupting Chemicals and Couples’ Pregnancy Outcomes: A Qualitative Review of the Literature

**DOI:** 10.1007/s40572-026-00550-w

**Published:** 2026-08-14

**Authors:** Han Han, Russ Hauser, Lidia Mínguez-Alarcón

**Affiliations:** 1 Departments of Nutrition, Harvard T.H. Chan School of Public Health, Boston, MA 02115 USA; 2 Department of Epidemiology, Harvard T.H. Chan School of Public Health, Boston, MA 02115 USA; 3 Department of Obstetrics, Gynecology and Reproductive Biology, Harvard Medical School, Boston, MA 02115 USA; 4 Department of Environmental Health, Harvard T.H. Chan School of Public Health, Boston, MA 02115 USA; 5 Channing Division of Network Medicine, Department of Medicine, Brigham and Women’s Hospital, Harvard Medical School, Boston, MA 02115 USA

**Keywords:** Endocrine disrupting chemicals, Paternal exposure, Preconception period, Pregnancy outcomes, Reproductive and birth outcomes

## Abstract

**Purpose of Review:**

Endocrine-disrupting chemicals (EDCs) are ubiquitous, and exposure to them has been associated with adverse reproductive health in both animals and humans. While the adverse effects of maternal EDC exposures on pregnancy outcomes have been extensively investigated, an increasing body of literature is now examining the role of paternal exposure, given its potential impact on sperm epigenetics. This review summarizes human studies examining associations between paternal exposure to several EDCs and couples’ pregnancy outcomes.

**Recent Findings:**

Biomarker-based studies suggest potential negative associations between paternal preconception exposure to certain phthalates and couples’ pregnancy outcomes, whereas studies for bisphenol A and parabens have shown little to no associations. The evidence regarding benzophenones, per- and polyfluoroalkyl substances, and flame-retardant chemicals remains limited and inconclusive.

**Summary:**

Given the critical knowledge gaps in this field, additional couple-based studies with repeated preconception exposure measurements are needed to clarify the effects of paternal exposure to individual EDCs and their mixtures on couples’ pregnancy outcomes. Additional studies on potential biological mechanisms that may explain these associations (e.g., sperm epigenome) are also warranted.

## Introduction

Adverse pregnancy outcomes, including hypertensive disease of pregnancy, fetal growth restriction, preterm delivery, and placental abruption, are common in Western populations [1–3] and have been increasingly recognized as contributors to long-term risks of chronic diseases and mortality in both mothers and their offspring [4–8]. Addressing modifiable barriers is therefore imperative to optimize the health of pregnant individuals and their fetuses. Accordingly, the American College of Obstetricians and Gynecologists has recommended reducing prenatal exposure to environmental chemicals, given their widespread presence and potential for reproductive and developmental toxicity [9]. However, most research to date has focused on evaluating the chemical effects of maternal exposures [10], while the potential impact of paternal exposures has largely been overlooked. Interestingly, a growing body of evidence indicates that paternal exposure to endocrine-disrupting chemicals (EDCs) is associated with reduced sperm quality parameters, increased sperm DNA damage, and epigenetic alterations [11, 12], which may in turn affect fertilization, embryonic development, and ultimately pregnancy outcomes [13, 14]. In this review, we summarize the available epidemiological literature studying the association between paternal exposures to EDCs and couples’ pregnancy outcomes.

## Methods

We conducted a narrative literature review of observational studies that evaluated associations between paternal exposure to selected EDCs and couples’ pregnancy outcomes. Our review focused on six classes of EDCs, including phthalates, bisphenol A (BPA), parabens, benzophenones, per- and polyfluoroalkyl substances (PFAS), and flame-retardant chemicals, given their high detection rates in Western populations [15–25]. From June to August 2025, we searched PubMed and Google Scholar using combinations of the following keywords: “paternal”, “pregnancy outcome”, “phthalates”, “bisphenol”, ‘BPA”, “paraben”, “benzophenone”, “PFAS”, “PBB”, “PBDE”, and “flame retardant”. We limited the search to human studies, English-language articles, and papers published within the past 15 years. We included peer-reviewed observational studies that: (1) assessed paternal exposure to at least one of the selected EDCs using biomarkers; and (2) reported at least one of the couples’ pregnancy outcomes (e.g., time-to-pregnancy, fertilization, embryo development, implantation, clinical pregnancy, live birth, gestational age, preterm birth, birth weight, birth size, secondary sex ratio). One reviewer (H.H.) performed the initial title- and abstract-level screening to identify relevant articles, and a second reviewer (L.M.-A.) verified potential inclusions. We also screened reference lists of included studies and conducted forward citation searches of key articles to identify additional eligible studies. In total, we included 18 studies on phthalates, 11 on BPA, 9 on parabens, 6 on benzophenones, 8 on PFAS, and 7 on flame-retardant chemicals in this review.

## Results

### Phthalates

Phthalates are synthetic chemicals widely used as plasticizers, solvents, or additives in a variety of consumer products, including food packaging, building materials, personal care products, and medical devices [26]. Phthalate exposure is widespread because these chemicals can readily leach, migrate, or off-gas from consumer products into the environment [27], with diet serving as a primary exposure source for certain phthalates [28]. Over the past two decades, human biomonitoring studies have consistently detected phthalate metabolites in urine samples from populations in North America [15–17], Europe [18], and Asia [29]. Extensive evidence from in vivo, in vitro, and epidemiological studies has demonstrated the reproductive toxicity of phthalates. As summarized in previous reviews of animal and human studies, exposure to certain phthalates is associated with impaired ovarian function, abnormal uterus development, hormone dysregulation, and adverse pregnancy outcomes in females [30], as well as reduced semen quality, testosterone suppression, and sperm DNA damage in males [11].

In our review, four epidemiological studies have examined paternal phthalate exposure in relation to couples’ fecundity as measured by time-to-pregnancy (Table 1) [31–34]. Of these, two are from the Longitudinal Investigation of Fertility and the Environment (LIFE) study with a prospective design but different biospecimen matrices (spot urine [31] vs. seminal plasma [34]); one uses a nested case-control design [32], and one is retrospective [33]. Only two studies accounted for potential confounding by maternal exposure, and both reported that higher urinary concentrations of certain phthalate metabolites in male partners were associated with longer time-to-pregnancy. Specifically, among 424 couples in the LIFE study [31], the multivariable-adjusted fecundability odds ratios (FORs; values < 1 indicate reduced fecundity) per 1-SD increase in log-transformed paternal preconception urinary concentrations were 0.81 (95% CI: 0.70, 0.94) for monomethyl phthalate (MMP) and 0.80 (95% CI: 0.67, 0.97) for monobenzyl phthalate (MBzP). Although the association for mono(2-carboxymethylhexyl) phthalate (MCMHP) in this study was only marginally significant (FOR: 0.86, 95% CI: 0.74,1.01) [31], a separate Chinese study found that paternal MCMHP exposure was related to reduced couples’ fecundity in the context of phthalate mixtures [32].

**Table 1:** Epidemiological studies of paternal phthalate exposure and couples’ pregnancy outcomes

	Location	Study design	Study population	Sample collection	Metabolites	Outcome	Model adjustment		Association findings (paternal exposure)
							Covariates	Adj. for female partner’s exposure	
Buck Louis et al. 2014 [31]	Michigan/Texas, USA	Prospective	424 couples discontinuing contraception and attempting to become pregnant	Single spot urine at enrollment (2005–2009)	MCPP, MMP, MEP, MiBP, MBP, MECPP, MCMHP, MEOHP, MEHHP, MCHP, MBzP, MEHP, MiNP, MOP (*n* = 14)	TTP	Age (F), age difference between partners, research site, BMI (F&M), serum cotinine (F&M), urinary creatinine (F&M), phthalate metabolite concentrations (F)	Y	↑MMP, MBzP ↑TTP
Yin et al. 2024 [32]	Shanghai, China	Nested case-control	107 couples with TTP > 12 menstrual cycles; 144 randomly selected couples with TTP ≤ 12 menstrual cycles	Single spot urine at enrollment (2016–2021)	MEP, MBP, MECPP, MCMHP, MEOHP, MEHHP, MEHP (*n* = 7); creatinine-corrected concentrations	Subfecundity	Age (M), BMI (M), education level (M), smoking (M), alcohol consumption (M), annual household income, home/office renovation within two years, exposure to rubber products	N (individual metabolite analyses) Y (couple-level mixture analyses)	Individual metabolite analyses: ↑MEHP, MCMHP, ∑DEHP, ∑PAEs ↑couples’ subfecundity Couple-level mixture analyses: ↑Mixture (e.g., male MCMHP) ↑couples’ subfecundity
Specht et al. 2015 [33]	Greenland Warsaw, Poland Kharkiv, Ukraine	Retrospective	160 male partners from couples in Greenland, 146 in Poland, 95 in Ukraine	Single serum at enrollment (2002–2004)	Secondary oxidized metabolites of DEHP: MEHHP, MEOHP, MECPP; diisononyl phthalate metabolites: MHINP, Mono(4-methyl-7-oxo-octyl) phthalate, MCNP	TTP	Age (F&M), BMI (M)	N	↑Proxy-MEHP: ↓TTP (Greenland), no association (Poland, Ukraine) ↑Proxy-MiNP: ↓TTP (Greenland), no association (Poland, Ukraine)
Buck Louis et al. 2018 [34]	Michigan/Texas, USA	Prospective	333 male partners from couples	Single seminal plasma at ~ 1 month after enrollment (2005–2009)	MCPP, MMP, MEP, MiBP, MBP, MECPP, MCMHP, MEOHP, MEHHP, MCHP, MBzP, MiNP, MOP, MHxP, mono-(8-methyl-1-nonyl) phthalate (*n* = 15)	TTP	Age (F), age difference between partners, research site, BMI (F&M), serum cotinine (F&M)	N	No association
Wu et al., 2016 [35]	Springfield, MA, USA	Prospective	50 couples undergoing IVF	Single spot urine on day of semen collection/oocyte retrieval (2014~)	MCPP, MMP, MEP, MiBP, MBP, MECPP, MEOHP, MEHHP, MBzP, MiNP, MEHP, MCNP, MCOP, MHBP, MHiBP (*n* = 15); phthalate alternatives: MCOCH, MHiNCH (*n* = 2); SG-corrected concentrations	Fertilization Embryo quality	Age (F&M), infertility status (M), phthalate metabolite concentrations (F), cleavage-stage embryo grade (for analyses of fertilization/blastocyst embryo quality)	Y	No association with fertilization ↑MEP ↑high-quality embryos (cleavage stage) ↑MBzP, MBP, MHBP, MMP ↓high-quality embryos (blastocyst stage) ↑MHBP, MMP, MCOCH ↓transferrable quality embryos (blastocyst stage)
Yang et al., 2023 [39]	Guangdong, China	Prospective	157 male partners undergoing IVF	Single spot urine collected at the female partner’s oocyte retrieval day (2020–2021)	MMP, MEP, MiPrP, MBP, MCHP, MBzP, MEHP, MHINP, MCNP, MCMHP, MEOHP, MEHHP, MECPP, MCPP, mono-2-heptyl phthalate (*n* = 15); SG-corrected concentrations Mixture (phthalates, phenols, organophosphate esters)	Fertilization Embryo quality	Age (M), BMI (M), smoking (M), passive smoking (M), alcohol drinking (M), education level (M), household income (M), infertility diagnosis (M), insemination technique (M), history of fathering a pregnancy (M)	N	No association with fertilization/high-quality blastocysts ↑MEOHP ↓MHINP ↓high-quality embryos ↑MEHP ↓blastocyst formation No association for paternal mixture exposure
Dodge et al., 2015 [36]	Boston, MA, USA	Prospective	218 couples undergoing 406 cycles (211 IVF, 195 IUI)	Spot urine collected at the female partner’s oocyte retrieval/insemination day for each treatment cycle (2004–2012)	MCPP, MEP, MiBP, MBP, MECPP, MEOHP, MEHHP, MBzP, MEHP, MCNP, MCOP (*n* = 11); SG-corrected concentrations	Fertilization Embryo quality Implantation Live birth	Age (F&M), total cycle number (F), BMI (F&M), smoking (F&M), phthalate metabolite concentrations (F)	Y	Among couples undergoing IVF: No trend for fertilization rate or high-quality embryos ↑MCOP, MCPP ↓odds of implantation ↓odds of live birth Among couples undergoing IUI: MBP (2nd/3rd vs. 1st quartile) ↓odds of live birth
Begum et al., 2021 [37]	San Francisco, CA, USA	Prospective	36 couples undergoing IVF	Single spot urine on day of semen collection/oocyte retrieval (2015–2016)	MEP, MBP, MECPP, MEHHP, MCHP, MBzP, MEHP, MiNP, MHxP, MPP, MiDP (*n* = 11)	Implantation Live birth	Age (F&M), urinary SG (F&M), histories of smoking (F&M), BMI (F), phthalate metabolite concentrations (F)	Y	↑MEHP ↓probability of implantation ↑MHxP ↓probability of live birth
Mínguez-Alarcón et al., 2021 [38]	Boston, MA, USA	Prospective	210 couples undergoing 300 IVF cycles	Spot urine collected at the female partner’s oocyte retrieval/insemination day for each treatment cycle (2004–2017)	Mixture components: MEP, MiBP, MBP, MECPP, MEOHP, MEHHP, MBzP, MEHP, bisphenol A, methylparaben, propylparaben	Live birth	Age (F&M), BMI (F&M), year of urine collection, urinary SG (F&M), infertility diagnosis, the other two mixture factors (M), female partner’s mixture factor (F)	Y	↑DEHP factor ↑risk of cycle failure before live birth ↑Bisphenol A/non-DEHP factor ↑risk of cycle failure prior to live birth (marginally significant)
Smarr et al., 2015 [40]	Michigan/Texas, USA	Prospective	233 couples with singleton live births	Single spot urine at enrollment (2005–2009)	MCPP, MMP, MEP, MiBP, MBP, MECPP, MCMHP, MEOHP, MEHHP, MCHP, MBzP, MEHP, MiNP, MOP (*n* = 14)	Gestational age Birth weight Birth length Head circumference	Age (M), race (M), education (M), urinary creatinine (F&M), BMI (M), serum cotinine (F&M), alcohol (M), conditional parity (F), phthalate metabolite concentrations (F), infant gender, chemical*gender	Y	↑MCHP, MECPP ↑gestational age MEOHP (2nd/3rd vs. 1st quartile) ↑gestational age ↑MCHP ↑birth weight No trend for birth length or head circumference
Zhang et al., 2020 [41]	Boston, MA, USA	Prospective	228 subfertile couples with singleton live births	Spot urine at enrollment and on the female partner’s oocyte retrieval/insemination day for each treatment cycle (2005–2018)	MCPP, MEP, MiBP, MBP, MECPP, MEOHP, MEHHP, MBzP, MEHP, MCNP, MCOP (*n* = 11); phthalate alternatives: MCOCH, MHiNCH (*n* = 2); SG-corrected concentrations	Preterm birth	Age (F&M), BMI (F&M), smoking (F&M), education level (F), infertility treatment type, phthalate metabolite concentrations (F)	Y	No association
Zhang et al., 2021 [42]	Boston, MA, USA	Prospective	203 subfertile couples with singleton live births	Spot urine at enrollment and on the female partner’s oocyte retrieval/insemination day for each treatment cycle (2005–2018)	Mixture components: MCPP, MEP, MiBP, MBP, MECPP, MEOHP, MEHHP, MBzP, MEHP, MCNP, MCOP, bisphenol A, methylparaben, propylparaben, butylparaben; SG-corrected concentrations	Preterm birth	Couple-based model (F&M exposures): age (F), race (F), BMI (F), smoking (F), education level (F), infertility treatment type	Y	↑Couples’ chemical mixture ↑preterm birth Suggestive positive trend between paternal MEHP and preterm birth
Messerlian et al., 2017 [43]	Boston, MA, USA	Prospective	174 subfertile couples with singleton live births (105 IVF-conceived, 69 non-IVF conceived)	Spot urine at enrollment and on the female partner’s oocyte retrieval/insemination day for each treatment cycle (2005–2016)	MCPP, MEP, MiBP, MBP, MECPP, MEOHP, MEHHP, MBzP, MEHP, MCNP, MCOP (*n* = 11); SG-corrected concentrations	Birth weight	Age (F&M), BMI (F&M), smoking (F&M), education level (F), gestational age, infertility diagnosis, phthalate metabolite concentrations (F)	Y	Among IVF-conceived singletons: ↑ΣDEHP, MEHHP, MEOHP, MECPP, MBP, MiBP, MBzP ↓birth weight Among non-IVF conceived singletons: No association with birth weight
Zhang et al., 2022 [44]	Boston, MA, USA	Prospective	203 subfertile couples with singleton live births	Spot urine at enrollment and on the female partner’s oocyte retrieval/insemination day for each treatment cycle (2005–2018)	Mixture components: MCPP, MEP, MiBP, MBP, MECPP, MEOHP, MEHHP, MBzP, MEHP, MCNP, MCOP, bisphenol A, methylparaben, propylparaben, butylparaben; SG-corrected concentrations	Birth weight	Couple-based model (F&M exposures): age (F&M), race (F), BMI (F&M), smoking (F&M), education level (F), infertility treatment type	Y	↑Couple’s chemical mixture (e.g. male MBP) ↓birth weight
Zang et al., 2023 [45]	Jiangsu, China	Prospective	544 ART and 940 spontaneous conception couples with singleton live births	Single spot urine collected on the day of semen collection/oocyte retrieval (ART-treated couples, 2015–2019) or at 8–14 weeks of gestation (spontaneously conceiving couples, 2014–2019)	MMP, MEP, MiBP, MHiBP, MBP, MHBP, MCPP, MBzP, MEHP, MEHHP, MEOHP, MECPP, MCMHP, MiNP, cx_MiNP, oxo_MiNP, oh_MiNP (*n* = 17); phthalate alternatives (*n* = 3); SG-corrected concentrations	Birth weight	Age (F&M), BMI (F&M), household income, infant sex, delivery mode (F), parity (F), smoking status (F&M), phthalate metabolite concentrations (F), infertility diagnosis and duration of infertility (ART-treated couples only)	Y	ART-treated couples: ↑MBP, MiBP, MHBP, MCPP, MBzP, MEHP, MEHHP ↓risk of low birth weight (< 2500 g) No association for paternal mixture exposure
Change et al., 2025 [46]	Goyang, South Korea	Prospective	73 mother-newborn-father triads	Single spot urine at delivery visit (2013–2017)	MEHP, MBP, MiBP, MBzP, MCHP, MEHHP, MEOHP, MECPP, MCMHP, MOP, MiNP, MCPP, MiDP, MEP, MMP, MPP (*n* = 16); creatinine-corrected concentrations	Placental weight Birth weight	Age (F), parity (F), pre-pregnancy BMI (F), pregnancy weight gain (F), gestational weeks (F), passive smoking (F), infant sex	N	No association for birth weight/placental weight
Mustieles et al., 2019 [47]	Boston, MA, USA	Prospective	68 subfertile couples with singleton live births	Spot urine at enrollment and on the female partner’s oocyte retrieval/insemination day for each treatment cycle (2005–2016)	MCPP, MEP, MiBP, MBP, MECPP, MEOHP, MEHHP, MBzP, MEHP, MCNP, MCOP (*n* = 11); SG-corrected concentrations	Placental weight	Age (F&M), BMI (F&M), smoking (F&M), education level (F), and infant sex	N	↑ΣDEHP, MECPP ↓placental weight No association with birth weight: placental weight ratio
Bae et al., 2015 [48]	Michigan/ Texas, USA	Prospective	205 couples with singleton live births	Single spot urine at enrollment (2005–2009)	MCPP, MMP, MEP, MiBP, MBP, MECPP, MCMHP, MEOHP, MEHHP, MCHP, MBzP, MEHP, MiNP, MOP (*n* = 14)	Secondary sex ratio	Couple-based model (F&M exposures): age (F), age difference between partners, annual income, research site, urinary creatinine (F&M), parity (F)	Y	↑MiBP ↓relative risk of a male birth

*Abbreviations*: *ART* Assisted reproductive technology, *BMI* Body mass index, *DEHP* di(2-ethylhexyl) phthalate, *F* Female partner, *IUI* Intrauterine insemination, *IVF* In vitro fertilization, *M* Male partner, *MBP* mono-n-butyl phthalate, *MBzP* monobenzyl phthalate, *MCHP* mono-cyclohexyl phthalate, *MCMHP* mono(2-carboxymethylhexyl) phthalate, *MCOCH* cyclohexane-1,2-dicarboxylic mono carboxyisooctyl ester, *MCOP* monocarboxyisooctyl phthalate, *MCPP* mono(3-carboxypropyl) phthalate, *MCNP* mono-carboxy-isononyl phthalate, *MECPP* mono(2-ethyl-5-carboxypentyl) phthalate, *MEHHP* mono(2-ethyl-5-hydroxyhexyl) phthalate, *MEP* monoethyl phthalate, *MEHP* mono(2-ethylhexyl) phthalate, *MEOHP* mono(2-ethyl-5-oxohexyl) phthalate, *MHBP* mono-3-hydroxybutyl phthalate, *MHiBP* mono-hydroxy-isobutyl phthalate, *MHiNCH* cyclohexane-1,2-dicarboxylic mono hydroxyisononyl ester, *MHINP* mono(hydroxy-isononyl) phthalate, *MHxP* monohexyl phthalate, *MiBP* mono-isobutyl phthalate, *MiDP* mono-isodecyl phthalate, *MiNP* mono-isononyl phthalate, *MiPrP* mono‐isopropyl phthalate, *MMP* monomethyl phthalate, *MOP* monooctyl phthalate, *MPP* mono-n-pentyl phthalate, *PAE* Phthalates, *SG* Specific gravity, *TTP* Time to pregnancy

Four [35–38] of five epidemiological studies of couples seeking fertility treatment found that paternal preconception exposure to specific phthalates was associated with early reproductive outcomes after accounting for maternal exposure (Table 1) [35–39]. Among 50 couples undergoing in vitro fertilization (IVF) in the Sperm Environmental Epigenetics and Development Study (SEEDS), Wu et al. reported that higher paternal urinary concentrations of MMP, MBzP, mono-3-hydroxybutyl phthalate (MHBP), and mono-n-butyl phthalate (MBP) were associated with decreased blastocyst quality, whereas monoethyl phthalate (MEP) was positively associated with high-quality embryos at the cleavage stage [35]. Yet, in the Environment and Reproductive Health (EARTH) Study, no clear trends were observed between male partners’ urinary phthalate metabolite concentrations and fertilization or embryo quality [36]. While the two available prospective studies suggest that paternal exposure to certain phthalates may adversely affect implantation [36, 37], the specific phthalate metabolites implicated differ between studies. For example, in the EARTH study of 152 couples undergoing 211 IVF cycles [36], Dodge et al. reported that male partners’ urinary concentrations of monocarboxyisooctyl phthalate (MCOP) and mono(3-carboxypropyl) phthalate (MCPP) were inversely associated with the odds of implantation, whereas another pilot study of 36 couples undergoing IVF identified mono(2-ethylhexyl) phthalate (MEHP) as the relevant metabolite [37]. Similarly, findings for live birth are inconsistent: Dodge et al. identified paternal MCOP and MCPP as inversely associated with the probability of live birth [36], whereas Begum et al. reported an inverse association with monohexyl phthalate (MHxP) [37]. Another EARTH publication of 210 couples undergoing 300 IVF cycles found that a paternal mixture exposure primarily driven by di(2-ethylhexyl) phthalate (DEHP) metabolites was associated with decreased probability of live birth; the multivariable-adjusted hazard ratio for cycle failure prior to live birth per interquartile range increase in the DEHP-related factor score was 1.38 (95% CI: 1.01, 1.89) [38]. The limited sample sizes, the narrow overlap in phthalate metabolites assessed, and the differing modeling approaches across available studies make it difficult to directly compare effect estimates.

As shown in Table 1, only one study has examined paternal preconception exposure to phthalates in relation to gestational age [40]. It reported that higher paternal urinary concentrations of mono(2-ethyl-5-carboxypentyl) phthalate (MECPP) and mono-cyclohexyl phthalate (MCHP) were associated with greater gestational age, independent of maternal exposure. The mean differences in gestation age between the highest and the lowest quartiles of paternal urinary concentrations were 6.4 days (95% CI: 0.3, 12.5) for MECPP and 7.0 days (95%CI: 2.2, 11.9) for MCHP (both *P* for trend < 0.05). For mono(2-ethyl-5-oxohexyl) phthalate (MEOHP), gestational age was greater in the second (*β* = 7.2, 95% CI: 2.4, 12.1) and third quartiles (*β* = 5.1, 95% CI: 0.02, 10.3) compared with the lowest quartile, although no monotonic trend was observed (*P* for trend = 0.22). With respect to preterm birth, the two available publications from the EARTH cohort reported no significant association with paternal phthalate exposure [41, 42]. As for birth weight, associations with paternal phthalate exposure reported across five studies varied by study population and exposure window [40, 43–46]. For example, paternal preconception urinary concentrations of certain phthalate metabolites, including DEHP metabolites such as mono(2-ethyl-5-hydroxyhexyl) phthalate (MEHHP), MEOHP, and MECPP, as well as MBP, mono-isobutyl phthalate (MiBP), and MBzP, were inversely associated with birth weight among IVF-conceived singletons, with metabolite-specific mean differences in birth weight per 1-unit increase in log-transformed concentrations ranging from − 139 to -92 g; no association was observed among non-IVF-conceived singletons [43]. Consistent inverse associations with birth weight were observed in subsequent analyses of the same EARTH cohort, in which phthalates and phenols were jointly modeled as paternal mixtures [44]. Conversely, Zang et al. found that higher paternal preconception urinary concentrations of several phthalate metabolites (MEHP, MEHHP, MiBP, MBzP, MHBP, MBP, and MCPP) were associated with a reduced risk of low birth weight (< 2500 g) among 554 assisted reproduction couples, whereas associations were largely null among 940 couples who conceived spontaneously with paternal exposure measured in the first trimester [45]. However, in the LIFE study of couples discontinuing contraception to conceive, paternal preconception urinary MCHP was positively associated with birth weight [40]. Another Korean study of 73 mother-newborn-father triads found no associations with birth weight for paternal phthalate metabolite concentrations measured at the delivery visit [46]. Regarding placental weight, paternal phthalate exposure during pregnancy showed no association [46], whereas preconception exposure was negatively associated [47]; however, neither analysis adjusted for maternal exposure. As for secondary sex ratio (SSR), the only available LIFE study reported that higher paternal preconception urinary MiBP concentrations were associated with fewer male births after adjustment for maternal exposure (per 1-SD increase in log-transformed urinary concentrations: relative risk [RR] = 0.82, 95% CI: 0.67, 1.00) [48].

Taken together, epidemiological evidence to date suggests that paternal preconception exposure to certain phthalates may be associated with adverse couples’ pregnancy outcomes, independent of maternal exposure. Negative associations with couples’ fecundity appear more consistent, whereas data for birth weight are mixed. However, findings for most endpoints are largely based on only one or two prospective studies and remain unconfirmed in other cohorts. The divergence in population characteristics (e.g., subfertile or general populations) and exposure patterns (e.g., levels and pathways of specific phthalates) may, in part, explain inconsistencies among existing studies. Although the molecular mechanisms linking paternal phthalate exposure with adverse pregnancy outcomes remain unclear, alterations in the sperm epigenome are thought to be contributing factors [12]. A recent meta-analysis of three preconception studies (EARTH, LIFE, and SEEDS) demonstrated that higher paternal urinary concentrations of several phthalate metabolites were associated with an increased number of sperm differentially methylated regions (DMRs), with MBzP, MiBP, and MEHP consistently identified across all cohorts [49]. As most of these DMRs were enriched in genes involved in spermatogenesis, hormone response and metabolism, embryonic organ development, and developmental growth, such sperm epigenetic modifications are likely linked to pregnancy outcomes [49]. Additional well-designed mechanistic and epidemiological studies are needed to confirm these associations.

### BPA

BPA is a high-production-volume synthetic chemical widely used in the manufacture of polycarbonate plastics and epoxy resins, as well as various plastic consumer products, including food and beverage containers, thermal paper receipts, toys, and dental sealants and composites [50]. Oral ingestion of contaminated food and beverages is the primary route of human exposure, while dermal absorption and inhalation from non-dietary sources also contribute [51], resulting in the widespread presence of BPA across populations [15–17, 19–21]. In April 2023, the European Food Safety Authority (EFSA) concluded that BPA likely causes reproductive toxicity in both females and males, based on animal and human evidence, and established a new tolerable daily intake (TDI) of 0.2 ng BPA/kg body weight (bw) per day for Europe, which is much lower than the previous TDI of 4 µg/kg bw per day set in 2015 [52]. Over the past few decades, many “BPA-free” alternatives, such as bisphenol S (BPS), have been introduced to the market [53]. Human biomonitoring studies indicate that urinary BPA concentrations have declined in populations from many countries over time, whereas BPS concentrations have increased [15, 19, 21]. However, animal and human studies show that BPS can disrupt sex hormones similarly to BPA [54], and may affect oocyte health even at low exposure levels [55].

Most epidemiological evidence on couples’ pregnancy outcomes to date has focused on paternal exposure to BPA (Table 2), with few studies examining the effect of BPA substitutes. Only two epidemiological studies have prospectively examined associations with couples’ fecundity, both from the LIFE cohort [31, 34]. Of these, one assessed BPA concentrations in spot urine samples [31] and the other measured seminal plasma levels of BPA, BPS, and bisphenol F [34], but neither study found an association. There is little evidence of an association between paternal urinary BPA or its substitute concentrations and fertilization rate or embryo quality among subfertile couples undergoing IVF treatment [39, 56]. For live birth, BPA showed no association when modeled individually [56], but a suggestive negative association was found when BPA was modeled as part of a mixture with non-DEHP metabolites [38]. Specifically, in the EARTH study of 210 couples undergoing 300 IVF cycles, the hazard ratio for cycle failure prior to live birth per interquartile range increase in the BPA/non-DEHP-related factor score was 1.26 (95% CI: 0.96, 1.65) after adjustment for maternal exposure and other confounders [38]. Also, in the EARTH study, no association was observed between paternal preconception urinary BPA concentrations and preterm birth [42, 57]. Two prospective studies examined the influence of paternal exposure to BPA on birth weight and head circumference and reported no associations independent of maternal exposure [40, 44, 58]. One study of 233 couples reported a positive association between paternal exposure to BPA and birth length [40]. Compared with the lowest quartile (≤ 0.23 ng/mL), the highest quartile (≥ 1.14 ng/mL) of paternal urinary BPA was associated with longer birth length (*β* = 1.35 cm, 95% CI: 0.25, 2.45; *P* for trend = 0.02) after adjustment for maternal BPA. The only available study on paternal BPA and the secondary sex ratio, including 205 couples with singleton live births from the LIFE cohort, reported that each 1-SD increase in log-transformed urinary BPA was associated with a 23% lower risk of male birth (RR: 0.77, 95% CI: 0.62, 0.95) in couple-based models with mutual adjustment for maternal and paternal exposure [48].

**Table 2:** Epidemiological studies of paternal exposure to BPA, parabens, and benzophenones in relation to couples’ pregnancy outcomes

	Location	Study design	Study population	Sample collection	Metabolites	Outcome	Model adjustment		Association findings (paternal exposure)
							Covariates	Adj. for female partner’s exposure	
BPA									
Buck Louis et al. 2014 [31]	Michigan/ Texas, USA	Prospective	424 couples discontinuing contraception and attempting to become pregnant	Single spot urine at enrollment (2005–2009)	BPA	TTP	Age (F), age difference between partners, research site, BMI (F&M), serum cotinine (F&M), urinary creatinine (F&M), BPA concentrations (F)	Y	No association
Buck Louis et al. 2018 [34]	Michigan/ Texas, USA	Prospective	339 male partners from couples	Single seminal plasma at ~ 1 month after enrollment (2005–2009)	BPA, BPF, BPS	TTP	Age (F), age difference between partners, research site, BMI (F&M), serum cotinine (F&M)	N	No association
Dodge et al., 2015 [56]	Boston, MA, USA	Prospective	218 couples undergoing 406 cycles (211 IVF, 195 IUI)	Spot urine collected at the female partner’s oocyte retrieval/insemination day for each treatment cycle (2004–2012)	BPA; SG-corrected concentrations	Fertilization Embryo quality Implantation Live birth	All models were adjusted for age (F). Other covariates, including age (M), BMI (F&M), and smoking (F&M), were included according to model fit.	N	No association with fertilization, implantation, or live birth No trend with high-quality embryos
Yang et al., 2023 [39]	Guangdong, China	Prospective	157 male partners undergoing IVF	Single spot urine collected at the female partner’s oocyte retrieval day (2020–2021)	BPA, BPE, BPB, BPM, BPG, BPS, BPAP, BPBP, BPPH (*n* = 9); SG-corrected concentrations Mixture (phthalates, phenols, organophosphate esters)	Fertilization Embryo quality	Age (M), BMI (M), smoking (M), passive smoking (M), alcohol drinking (M), education level (M), household income (M), infertility diagnosis (M), insemination technique (M), history of fathering a pregnancy (M)	N	No association
Mínguez-Alarcón et al., 2021 [38]	Boston, MA, USA	Prospective	210 couples undergoing 300 IVF cycles	Spot urine collected at the female partner’s oocyte retrieval/insemination day for each treatment cycle (2004–2017)	Mixture components: MEP, MiBP, MBP, MECPP, MEOHP, MEHHP, MBzP, MEHP, BPA, MeP, PrP	Live birth	Age (F&M), BMI (F&M), year of urine collection, urinary SG (F&M), infertility diagnosis, the other two mixture factors (M), female partner’s mixture factor (F)	Y	↑BPA/non-DEHP factor ↑risk of cycle failure prior to live birth (marginally significant)
Mustieles et al., 2020 [57]	Boston, MA, USA	Prospective	228 subfertile couples with singleton live births	Spot urine at enrollment and on the female partner’s oocyte retrieval/insemination day for each treatment cycle (2005–2018)	BPA; SG-corrected concentrations	Preterm birth	Age (F&M), BMI (F&M), smoking (F&M), education level (F), infertility treatment type, preconception BPA concentrations (F)	Y	No association
Zhang et al., 2021 [42]	Boston, MA, USA	Prospective	203 subfertile couples with singleton live births	Spot urine at enrollment and on the female partner’s oocyte retrieval/insemination day for each treatment cycle (2005–2018)	Mixture components: MCPP, MEP, MiBP, MBP, MECPP, MEOHP, MEHHP, MBzP, MEHP, MCNP, MCOP, BPA, MeP, PrP, BuP; SG-corrected concentrations	Preterm birth	Couple-based model (F&M exposures): age (F), race (F), BMI (F), smoking (F), education level (F), infertility treatment type	Y	↑Couples’ chemical mixture ↑preterm birth No trend between paternal BPA and preterm birth
Smarr et al., 2015 [40]	Michigan/ Texas, USA	Prospective	233 couples with singleton live births	Single spot urine at enrollment (2005–2009)	BPA	Gestational age Birth weight Birth length Head circumference	Age (M), race (M), education (M), urinary creatinine (F&M), BMI (M), serum cotinine (F&M), alcohol (M), conditional parity (F), BPA concentrations (F), infant gender, chemical*gender	Y	↑BPA **↑**birth length No association with gestational age, birth weight, or head circumference
Mustieles et al., 2018 [58]	Boston, MA, USA	Prospective	166 subfertile couples with singleton live births	Spot urine at enrollment and on the female partner’s oocyte retrieval/insemination day for each treatment cycle (2005–2016)	BPA; SG-corrected concentrations	Birth weight Head circumference	Age (F&M), BMI (F&M), smoking (F&M), education level (F), infertility treatment type, prenatal BPA concentrations (F)	Y	No association
Zhang et al., 2022 [44]	Boston, MA, USA	Prospective	203 subfertile couples with singleton live births	Spot urine at enrollment and on the female partner’s oocyte retrieval/insemination day for each treatment cycle (2005–2018)	Mixture components: MCPP, MEP, MiBP, MBP, MECPP, MEOHP, MEHHP, MBzP, MEHP, MCNP, MCOP, BPA, MeP, PrP, BuP; SG-corrected concentrations	Birth weight	Couple-based model (F&M exposures): age (F&M), race (F), BMI (F&M), smoking (F&M), education level (F), infertility treatment type	Y	↑Couple’s chemical mixture ↓birth weight (paternal BPA was not the main contributor)
Bae et al., 2015 [48]	Michigan/ Texas, USA	Prospective	205 couples with singleton live births	Single spot urine at enrollment (2005–2009)	BPA	Secondary sex ratio	Couple-based model (F&M exposures): age (F), age difference between partners, annual income, research site, urinary creatinine (F&M), parity (F)	Y	↑BPA ↓relative risk of a male birth
Parabens									
Smarr et al., 2017 [64]	Michigan/Texas, USA	Prospective	501 couples discontinuing contraception and attempting to become pregnant	Single spot urine at enrollment (2005–2009)	MeP, PrP, EtP, BuP, BzP, HeP (*n* = 6)	TTP	Age (F), age difference between partners, research site, race (F&M), income (F&M), BMI (F&M), serum cotinine (F&M), urinary creatinine (F&M), paraben metabolite concentrations (F)	Y	No association
Ao et al. 2023 [65]	Shanghai, China	Prospective	884 couples attempting to become pregnant	Single spot urine at enrollment (2013–2015)	MeP, PrP, EtP, BuP, BzP, HeP (*n* = 6)	TTP	Couple-based model (F&M exposures): age (F), age difference between partners, BMI (F&M), urinary creatinine (F&M), education level (F&M), smoking (F&M), alcohol consumption (F&M), parity (F), family income	Y	No association
Yang et al., 2023 [39]	Guangdong, China	Prospective	157 male partners undergoing IVF	Single spot urine collected at the female partner’s oocyte retrieval day (2020–2021)	MeP, BuP, BzP, EtP, PrP, HeP (*n* = 6); SG-corrected concentrations Mixture (phthalates, phenols, organophosphate esters)	Fertilization Embryo quality	Age (M), BMI (M), smoking (M), passive smoking (M), alcohol drinking (M), education level (M), household income (M), infertility diagnosis (M), insemination technique (M), history of fathering a pregnancy (M)	N	↑PrP, MeP ↓normal fertilization ↑PrP ↓high-quality embryos ↓blastocyst formation No association for paternal mixture exposure
Dodge et al., 2015 [56]	Boston, MA, USA	Prospective	218 couples undergoing 406 cycles (211 IVF, 195 IUI)	Spot urine collected at the female partner’s oocyte retrieval/insemination day for each treatment cycle (2004–2012)	MeP, PrP, BuP; SG-corrected concentrations	Fertilization Embryo quality Implantation Live birth	All models were adjusted for age (F). Other covariates, including age (M), BMI (F&M), and smoking (F&M), were included according to model fit.	N	No association for any IVF outcomes MeP (2nd vs. 1st quartile) ↓live birth following IUI (no trend observed) ↑PrP ↓live birth following IUI
Mínguez-Alarcón et al., 2021 [38]	Boston, MA, USA	Prospective	210 couples undergoing 300 IVF cycles	Spot urine collected at the female partner’s oocyte retrieval/insemination day for each treatment cycle (2004–2017)	Mixture components: MEP, MiBP, MBP, MECPP, MEOHP, MEHHP, MBzP, MEHP, BPA, MeP, PrP	Live birth	Age (F&M), BMI (F&M), year of urine collection, urinary SG (F&M), infertility diagnosis, the other two mixture factors (M), female partner’s mixture factor (F)	Y	No association
Mustieles et al., 2020 [57]	Boston, MA, USA	Prospective	228 subfertile couples with singleton live births	Spot urine at enrollment and on the female partner’s oocyte retrieval/insemination day for each treatment cycle (2005–2018)	MeP, PrP, BuP, EtP; SG-corrected concentrations	Preterm birth	Age (F&M), BMI (F&M), smoking (F&M), education level (F), infertility treatment type, preconception biomarker concentrations (F)	Y	↑∑Parabens (the molar sum of PrP, BuP, MeP) ↑preterm birth (marginally significant)
Zhang et al., 2021 [42]	Boston, MA, USA	Prospective	203 subfertile couples with singleton live births	Spot urine at enrollment and on the female partner’s oocyte retrieval/insemination day for each treatment cycle (2005–2018)	Mixture components: MCPP, MEP, MiBP, MBP, MECPP, MEOHP, MEHHP, MBzP, MEHP, MCNP, MCOP, BPA, MeP, PrP, BuP; SG-corrected concentrations	Preterm birth	Couple-based model (F&M exposures): age (F), race (F), BMI (F), smoking (F), education level (F), infertility treatment type	Y	↑Couples’ chemical mixture ↑preterm birth No trend between paternal parabens and preterm birth
Messerlian et al., 2018 [66]	Boston, MA, USA	Prospective	184 subfertile couples with singleton live births	Spot urine at enrollment and on the female partner’s oocyte retrieval/insemination day for each treatment cycle (2005–2016)	MeP, PrP, BuP, EtP; SG-corrected concentrations	Birth weight Head circumference	Age (F&M), BMI (F&M), smoking (F&M), education level (F), infertility treatment type, season, prenatal biomarker concentrations (F)	Y	No association
Zhang et al., 2022 [44]	Boston, MA, USA	Prospective	203 subfertile couples with singleton live births	Spot urine at enrollment and on the female partner’s oocyte retrieval/insemination day for each treatment cycle (2005–2018)	Mixture components: MCPP, MEP, MiBP, MBP, MECPP, MEOHP, MEHHP, MBzP, MEHP, MCNP, MCOP, BPA, MeP, PrP, BuP; SG-corrected concentrations	Birth weight	Couple-based model (F&M exposures): age (F&M), race (F), BMI (F&M), smoking (F&M), education level (F), infertility treatment type	Y	↑Couple’s chemical mixture ↓birth weight (paternal parabens were not the main contributors)
Benzophenones									
Buck Louis et al. 2018 [34]	Michigan/Texas, USA	Prospective	339 male partners from couples	Single seminal plasma at ~ 1 month after enrollment (2005–2009)	BP-1, BP-2, BP-3, BP-8, 4-OH-BP (*n* = 5)	TTP	Age (F), age difference between partners, research site, season, BMI (F&M), serum cotinine (M)	N	No association
Buck Louis et al. 2014 [71]	Michigan/Texas, USA	Prospective	424 couples discontinuing contraception and attempting to become pregnant	Single spot urine at enrollment (2005–2009)	5 benzophenone-type ultraviolet filters (i.e., BP-3, BP-1, BP-2, BP-8, 4-OH-BP)	TTP	Age (F), age difference between partners, research site, season, BMI (F&M), serum cotinine (F&M), urinary creatinine (F&M), UV filter concentrations (F)	Y	↑BP-2 ↑TTP
Yang et al., 2023 [39]	Guangdong, China	Prospective	157 male partners undergoing IVF	Single spot urine collected at the female partner’s oocyte retrieval day (2020–2021)	BP-1, BP-2, BP-3, BP-4, 4-OH-BP (*n* = 5); SG-corrected concentrations Mixture (phthalates, phenols, organophosphate esters)	Fertilization Embryo quality	Age (M), BMI (M), smoking (M), passive smoking (M), alcohol drinking (M), education level (M), household income (M), infertility diagnosis (M), insemination technique (M), history of fathering a pregnancy (M)	N	No association
Mustieles et al., 2020 [57]	Boston, MA, USA	Prospective	137 subfertile couples with singleton live births	Spot urine at enrollment and on the female partner’s oocyte retrieval/insemination day for each treatment cycle (2005–2018)	BP-3; SG-corrected concentrations	Preterm birth	Age (F&M), BMI (F&M), smoking (F&M), education level (F), infertility treatment type, preconception biomarker concentrations (F)	Y	No association
Messerlian et al., 2018 [66]	Boston, MA, USA	Prospective	101 subfertile couples with singleton live births	Spot urine at enrollment and on the female partner’s oocyte retrieval/insemination day for each treatment cycle (2005–2016)	BP-3; SG-corrected concentrations	Birth weight Head circumference	Age (F&M), BMI (F&M), smoking (F&M), education level (F), infertility treatment type, season, prenatal biomarker concentrations (F)	Y	↑BP-3 ↑birth weight No association with head circumference
Bae et al., 2016 [72]	Michigan/Texas, USA	Prospective	205 couples with singleton live births	Single spot urine at enrollment (2005–2009)	5 benzophenone-type ultraviolet filters (i.e., BP-3, BP-1, BP-2, BP-8, 4-OH-BP)	Secondary sex ratio	Couple-based model (F&M exposures): age (F&M), annual household income, research site, urinary creatinine (F&M), parity (F)	Y	↑BP-2 ↓relative risk of a male birth

*Abbreviations*: *4OH-BP* 4-hydroxy-benzophenone, *BMI* Body mass index, *BP-1* benzophenone-1, *BP-2* benzophenone-2, *BP-3* benzophenone-3, *BP-4* benzophenone-4, *BP-8* benzophenone-8, *BPA* bisphenol A, *BPAP* bisphenol AP, *BPB* bisphenol B, *BPBP* bisphenol BP, *BPE* bisphenol E, *BPF* bisphenol F, *BPG* bisphenol G, *BPM* bisphenol M, *BPPH* bisphenol PH, *BPS* bisphenol S, *BuP* butylparaben, *BzP* benzylparaben, *DEHP* di(2-ethylhexyl) phthalate, *EtP* ethylparaben, *F* Female partner, *HeP* heptylparaben, *IUI* Intrauterine insemination, *IVF* In vitro fertilization, *M* Male partner, *MBP* mono-n-butyl phthalate, *MBzP* monobenzyl phthalate, *MCOP* monocarboxyisooctyl phthalate, *MCPP* mono(3-carboxypropyl) phthalate, *MCNP* mono-carboxy-isononyl phthalate, *MECPP* mono(2-ethyl-5-carboxypentyl) phthalate, *MEHHP *mono(2-ethyl-5-hydroxyhexyl) phthalate, *MeP* methylparaben, *MEP* monoethyl phthalate, *MEHP* mono(2-ethylhexyl) phthalate, *MEOHP* mono(2-ethyl-5-oxohexyl) phthalate, *MiBP* mono-isobutyl phthalate, *PrP* propylparaben, *SG* Specific gravity, *TTP* Time to pregnancy

Altogether, there is a growing body of evidence examining the association between paternal exposure to BPA and couples’ pregnancy outcomes, yet most studies report null findings. However, the available evidence on specific outcomes is still too limited to draw firm conclusions about the influence of paternal BPA. In some studies (e.g., the LIFE study), only a single BPA measurement from a spot urine sample collected at baseline was used. Given the short half-lives of BPA, exposure misclassification may exist and bias results toward the null. However, several EARTH publications used multiple preconception urine samples collected at baseline and per IVF treatment cycle and still found no associations with birth outcomes. Notably, six of the 11 existing studies come from the EARTH cohort of subfertile couples. Thus, more diverse cohorts with repeated urinary measurements during the preconception window are needed before the role of paternal BPA exposure can be clarified.

### Parabens

Parabens, also known as para-hydroxybenzoic acid esters, are widely used as preservatives in cosmetics, personal care products, pharmaceuticals, food and beverage processing, and insect repellants due to their antibacterial and antifungal properties [59]. The most common types include methylparaben (MeP), butylparaben (BuP), propylparaben (PrP), and ethylparaben (EtP), which are often used as mixtures to enhance antimicrobial efficacy [60]. Human exposure to parabens occurs through dermal absorption, inhalation, and ingestion during the use of these products [61]. Although exposure to parabens has shown a decreasing trend over the past two decades [15–17], pregnant women and children are considered susceptible populations [62]. While the endocrine-disrupting properties of parabens are well-established in experimental studies, the effects of paraben exposure on human reproductive health remain less clear, given the limited number of epidemiological studies and their mixed findings [11, 63].

Only a handful of epidemiological studies have evaluated the association between paternal preconception paraben exposure and couples’ pregnancy outcomes, with seven out of nine such studies included subfertile populations seeking fertility care (Table 2). The remaining two studies from general populations examined couples’ fecundity, and both found that maternal, rather than paternal, paraben exposure was associated with longer time-to-pregnancy [64, 65]. Regarding IVF treatment outcomes, findings were inconsistent across the three available studies. In a study of 157 Chinese men, Yang et al. reported inverse associations of paternal urinary MeP and PrP with normal fertilization, as well as of PrP with embryo quality [39], whereas no such associations were observed in the EARTH study [56]. Notably, both studies modeled paternal paraben exposure individually and did not adjust for maternal paraben exposure [39, 56]. Another study that analyzed parabens as part of a mixture and accounted for the influence of maternal exposure found no association with live birth [38]. Also among subfertile couples in the EARTH study, Mustieles et al. observed a suggestive positive association between paternal urinary paraben concentrations and preterm birth. After adjustment for maternal preconception exposure, each natural log-unit increase in the molar sum of MeP, PrP, and BuP was associated with a RR of preterm birth (< 37 weeks) of 1.42 (95% CI: 0.97, 2.09) [57]. Yet, when modeled as part of a mixture with BPA and phthalates, paternal parabens did not show any trend with preterm birth risk [42]. No associations between paternal exposure to parabens and birth weight were found in either single-chemical [66] or mixture analyses [44].

Overall, there is some evidence that paternal paraben exposure may be associated with preterm birth; however, it remains unclear whether this association is causal or whether paraben exposure merely serves as a proxy of co-exposure to other chemicals, given the divergent results between single-chemical and mixture analyses. Additionally, the generalizability of these findings to broader populations from different cohorts requires confirmation.

### Benzophenones

Benzophenones (BPs) are chemicals consisting of two benzene rings linked by a carbonyl group and are widely used as active ultraviolet (UV) filters in sunscreens for their UV absorption properties [67]. BPs are also incorporated into cosmetics, personal care products, building materials, and plastic coatings to prevent photodegradation and enhance product durability [68]. Their extensive use leads to ubiquitous and sustained human exposure, primarily through dermal absorption, as well as inhalation and ingestion. Human biomonitoring studies indicate that benzophenone-3 (BP-3) is the most frequently detected BP congener in urine and blood, followed by its main metabolite benzophenone-1 (BP-1), while other congeners, such as 4-hydroxy-benzophenone (4-OH-BP) and benzophenone-2 (BP-2), are detected less often [69]. Accumulating evidence indicates that certain BPs, particularly BP-3, can affect female reproductive health, with limited findings for male reproductive toxicity at human-relevant concentrations [70].

Six epidemiological studies have investigated the associations between paternal BP exposure and couples’ pregnancy outcomes (Table 2). Two studies from the LIFE cohort suggest that paternal exposure to certain BPs may be associated with reduced couples’ fecundity [34, 71]. Higher paternal urinary BP-2, dichotomized at the 75^th^ percentile of the 424 male partners’ concentrations, was associated with longer time-to-pregnancy (FOR: 0.69, 95% CI: 0.50, 0.95) after adjustment for female partner’s concentrations and other confounders [71]. When BPs were measured in seminal plasma, BP-3 levels showed a marginally significant positive association with time-to-pregnancy among 339 male partners [34]. To our knowledge, there is no prospective evidence on paternal BP exposure and early reproductive outcomes that carefully accounts for maternal exposure. Evidence from the EARTH study showed that paternal urinary BP-3 concentrations were not associated with preterm birth [57] or infant’s head circumference [66], but were positively associated with birth weight [66]. After adjusting for maternal prenatal BP-3, each natural log-unit increase in paternal preconception BP-3 concentration was associated with a 153 g increase in birth weight (95% CI: 74, 234) among 93 subfertile couples with singleton live births. In addition, higher paternal BP-2 exposure was associated with fewer male births, independent of maternal exposure, among 205 couples with singleton births in the LIFE study [72]. The multivariable-adjusted RR of male births for the highest versus lowest tertile of paternal urinary BP-2 was 0.67 (95% CI: 0.45, 0.99; *P* for trend = 0.04).

In summary, paternal preconception exposure to certain BPs may be associated with couples’ fecundity, birth weight, and offspring sex ratio, although the current evidence is limited and comes primarily from single cohorts. As such, additional studies are needed to confirm these findings and to rule out the possibility that the observed association is due to chance.

### PFAS

PFAS, often referred to as “forever chemicals”, are a vast family of synthetic compounds characterized by carbon-fluorine bonds, which confer high resistance to degradation and heat as well as significant environmental persistence [73]. PFAS are widely used in the manufacture of consumer and industrial products, including plastics, rubbers, electronic devices, building materials, coatings, and paints [74]. Due to their ability to cycle in the hydrosphere, PFAS have become widespread contaminants on a global scale [75]. Although levels of certain legacy PFAS have declined over the past two decades, these substances remain detectable in blood samples across populations worldwide [23–25, 76]. Accumulating evidence from animal and human studies has linked PFAS exposure to female and male reproductive disorders [73] .

To date, eight epidemiological studies have examined the influence of paternal PFAS exposure on couples’ pregnancy outcomes (Table 3). Of these, seven used prospective or nested case-control designs [77–83], whereas one was retrospective [84]. Of the seven studies with prospective associations, five assessed preconception exposure [77–80, 83], and two measured exposure in mid- to late-pregnancy [81, 82]. Although certain PFAS have elimination half-lives of several years in the human body [25], paternal serum concentrations measured during pregnancy may not accurately reflect cumulative preconception exposure, which is necessary for assessing paternal effects on gestational processes independent of direct maternal exposure [12]. Findings on couples’ fecundity remain inconsistent, with available studies reporting null [77], positive [78], and negative [84] associations with selected PFAS. The inconsistency may be partly attributed to differences in study design (prospective [77, 78] vs. retrospective [84]), adjustment for maternal exposure (with [78] vs. without [77, 84]), and exposure patterns across countries and calendar periods. Only one study has examined paternal preconception PFAS exposure in relation to IVF treatment outcomes, suggesting inverse associations with fertilization and embryo quality but not with implantation, clinical pregnancy, or live birth [79]. Among 96 Chinese couples undergoing IVF, the highest tertile of paternal plasma perfluorooctanoate (PFOA; 42.0-81.4 ng/mL) was associated with a reduced number of two-pronuclei zygotes (*P* for trend = 0.047) and day 3 good-quality embryos (*P* for trend = 0.08) compared with the lowest tertile (6.0-16.1 ng/mL) [79]. In the EARTH study, no association was found between paternal preconception PFAS exposure and either gestational age or preterm birth [80]. However, preconception serum concentrations of PFOA, perfluorooctane sulfonate (PFOS), and perfluorohexane sulfonate (PFHxS) were associated with birth weight in opposite directions for fathers and mothers: birth weight increased with paternal exposure and decreased with maternal exposure [80]. In addition, the association for paternal PFOS was more pronounced among male infants. Another study of 224 couples in the LIFE cohort found that higher paternal preconception serum concentrations of N-methylperfluorooctane sulfonamidoacetic acid (MeFOSAA) were suggestively associated with fewer male births in a dose-response relationship [83]. Compared with the lowest tertile, the multivariable-adjusted odds ratios (ORs) for male births in the second tertile and the highest tertiles of paternal serum MeFOSAA were 0.53 (95% CI: 0.26, 1.10) and 0.34 (95% CI: 0.13, 0.89), respectively.

**Table 3:** Epidemiological studies of paternal exposure to PFAS and flame-retardant chemicals in relation to couples’ pregnancy outcomes

	Location	Study design	Study population	Sample collection	Metabolites	Outcome	Model adjustment		Association findings (paternal exposure)
							Covariates	Adj. for female partner’s exposure	
PFAS									
Buck Louis et al. 2013 [77]	Michigan/Texas, USA	Prospective	401 couples discontinuing contraception and attempting to become pregnant	Single serum at enrollment (2005–2009)	EtFOSAA, MeFOSAA, PFDA, PFNA, PFOSA, PFOS, PFOA (*n* = 7)	TTP	The sum of all other chemicals in the same chemical class (M), age (M), BMI (M), serum cotinine (M), research site	N	No association
Jørgensen et al. 2014 [84]	Greenland Warsaw, Poland Kharkiv, Ukraine	Retrospective	160 male partners from couples in Greenland, 146 in Poland, 95 in Ukraine	Single serum at enrollment (2002–2004)	PFOA, PFOS, PFHxS, PFNA (*n* = 4)	TTP	Age (F&M), BMI (M), country (in pooled analyses)	N	↑PFNA ↑TTP (Greenland) No association (Poland, Ukraine)
Luo et al. 2022 [78]	Shanghai, China	Prospective	936 couples attempting to become pregnant	Single plasma at enrollment (2013–2015)	Linear PFOA, PFNA, PFDA, PFUnDA, PFDoA, PFTrDA, PFTeDA, linear PFOS, PFHpS, PFHxS, 7 branched PFOS isomers, 2 branched PFOA isomers, 2 Cl-PFESAs, PFBA, PFHpA, PFBS, 6:2 diPAP (*n* = 25)	TTP	Couple-based model (F&M exposures): age (F), age difference between partners, BMI (F&M), education level (F&M), smoking (F&M), alcohol consumption (F&M), parity (F), family income	Y	↑n-PFOS, n-PFOA, PFHpS, PFHxS, 6:2Cl-PFESA ↓TTP
Ma et al., 2021 [79]	Zhejiang, China	Prospective	96 couples undergoing IVF	Single plasma on day of semen collection/oocyte retrieval (2017)	PFOA, PFOS, PFNA, PFDA, PFUnDA, PFHxS, PFDoA, PFHpA, PFOSA, PFBS (*n* = 10)	Fertilization Embryo quality Implantation Clinical pregnancy Live birth	Age (F&M), BMI (M), smoking (M), and preconception serum PFAS levels (F)	Y	↑PFOA ↓number of two-pronuclei zygotes ↑PFOA ↓number of good-quality embryos (suggestive trend) No associations with implantation, clinical pregnancy, and live birth
Zhang et al., 2024 [80]	Boston, MA, USA	Prospective	137 subfertile couples with singleton live births	Single serum at enrollment (serum; 2005–2019)	linear PFOA, sum of branched PFOA isomers, linear PFOS, sum of PFOS isomers, PFNA, PFHxS, PFDA, PFUnDA, MeFOSAA (*n* = 9)	Gestational age Preterm birth Birth weight	Couple-based model (F&M exposures): age (F&M), race (F), BMI (F&M), smoking (F&M), education level (F), parity (F), infertility treatment type, study period	Y	↑PFOA, PFOS, PFHxS ↑birth weight (more apparent in male infants) No associations with gestational age or preterm birth
Guo et al., 2025 [81]	Greenland Warsaw, Poland Kharkiv, Ukraine	Prospective	178 male partners from couples in Greenland, 142 in Poland, 178 in Ukraine	Single serum during mid- to late- pregnancy (2002–2004)	PFOA, PFOS, PFNA, PFDA, PFHxS, PFUnDA, PFDoA (*n* = 7)	Gestational age Birth weight Birth length	Study region, age (F), parental age difference, pre-pregnancy BMI (F&M), education (F), parity (F), infant sex, spousal exposure to the same type of PFAS (F)	Y	No associations with gestational age or birth weight ↑PFOS, PFNA, PFDA, PFHxS ↓birth length (marginally significant)
Yu et al., 2022 [82]	Guangdong, China	Nested case-control	355 singleton preterm births and 481 term births	Single serum during the third trimester (2015–2018)	PFOA, PFOS, PFNA, PFDA, PFHxS, PFHxA, PFBA (*n* = 7)	Preterm birth	Age (M), BMI (M), active smoke (M), alcohol intake during pregnancy (M), infant sex, family income, seafood consumption (M), parity (F)	N	↑PFHxA ↑preterm birth ↑PFHxS, PFOS, PFNA ↓preterm birth
Bae et al., 2015 [83]	Michigan/Texas, USA	Prospective	224 couples with singleton live births	Single serum at enrollment (2005–2009)	PFOS, PFOA, PFNA, PFDA, PFOSA, EtFOSAA, MeFOSAA (*n* = 7)	Secondary sex ratio	Couple-based model (F&M exposures): age (F&M), annual household income, research site, parity (F)	Y	MeFOSAA (3rd vs. 1st tertile) ↓probability of a male birth (suggestive trend)
Flame retardants									
Buck Louis et al. 2013 [77]	Michigan/Texas, USA	Prospective	401 couples discontinuing contraception and attempting to become pregnant	Single serum at enrollment (2005–2009)	PBB-153, PBDEs 17, 28, 47, 66, 85, 99, 100, 153, 154, 183	TTP	The sum of all other chemicals in the same chemical class (M), age (M), BMI (M), serum cotinine (M), serum lipids (M), research site	N	↑PBDE-183 ↑TTP (marginally significant)
Carignan et al., 2018 [90]	Boston, MA, USA	Prospective	201 couples undergoing 276 IVF cycles	Spot urine collected at the female partner’s oocyte retrieval/insemination day for each treatment cycle (2005–2015)	BCIPP, BDCIPP, DPHP, ip-PPP, and tb-PPP; SG-corrected concentrations	Fertilization Embryo quality Implantation Clinical pregnancy Live birth	Age (F&M), BMI (F&M), race (F&M), year of IVF treatment cycle, infertility diagnosis, organophosphate flame-retardant metabolite concentrations (F)	Y	BDCIPP (2nd/3rd vs. 1st quartile) ↓proportion of fertilization (suggestive trend) No association with embryo quality, implantation, clinical pregnancy, or live birth
Ingle et al., 2021 [89]	Boston, MA, USA	Prospective	189 couples undergoing 285 IVF cycles	Single serum at enrollment (2006–2016)	5 PBDE congeners: 47, 99, 100, 153, 154	Fertilization Implantation Clinical pregnancy Live birth	Age (F&M), serum lipids (F&M), BMI (F&M), year of serum sample collection, infertility diagnosis, serum PBDE concentrations (F)	Y	No association with fertilization rates No trend with implantation, clinical pregnancy, or live birth
Yang et al., 2023 [39]	Guangdong, China	Prospective	157 male partners undergoing IVF	Single spot urine collected at the female partner’s oocyte retrieval day (2020–2021)	BCIPP, DPHP, BBOEP; SG-corrected concentrations Mixture (phthalates, phenols, organophosphate esters)	Fertilization Embryo quality	Age (M), BMI (M), smoking (M), passive smoking (M), alcohol drinking (M), education level (M), household income (M), infertility diagnosis (M), insemination technique (M), history of fathering a pregnancy (M)	N	↑DPHP ↓high-quality embryos
Redmond et al., 2022 [91]	Michigan, USA	Prospective	155 fathers matched to 336 offspring	Single serum collected at enrollment (1976–78)	PBB	Gestational age Birth weight	Offspring born to the same father, offspring gestational age and sex, age at offspring birth (M), age at PBB exposure (M), education level (M), and PBB concentrations (F)	Y	↑PBB ↑risk of low birthweight (< 3232 g) No associations with gestational age
Robledo et al., 2015 [92]	Michigan/ Texas, USA	Prospective	234 couples with singleton live births	Single serum at enrollment (2005–2009)	PBB-153, PBDEs 17, 28, 47, 66, 85, 99, 100, 153, 154, 183	Birth weight Birth length Head circumference	Serum lipids (F&M), serum cotinine (F&M), pre-pregnancy BMI (F), age (F), difference in parental age, infant sex, the individual and partner sum of remaining chemical concentrations in each chemical’s respective class	Y	↑PBDE-183 ↓birth weight among girls No associations with birth length or head circumference
Bae et al., 2018 [93]	Michigan/Texas, USA	Prospective	235 couples with singleton live births	Single serum at enrollment (2005–2009)	PBB-153, PBDEs 17, 28, 47, 66, 85, 99, 100, 153, 154, 183	Secondary sex ratio	Couple-based model (F&M exposures): serum lipid (F&M), age (F), difference in parental age, annual income, serum cotinine (F&M), the sum of remaining chemicals in the relevant class of compounds (F&M)	Y	No association

*Abbreviations*: *BBOEP* bis(2butoxyethyl) phosphate, *BCIPP* bis(1-chloro-2-propyl) phosphate, *BDCIPP* bis(1,3-dichloro-2-propyl) phosphate, *BMI* body mass index, *Cl: PFESAs* chlorinated polyfluoroalkyl ether sulfonic acids, *DPHP* diphenyl phosphate, *EtFOSAA* N-ethylperfluorooctane sulfonamidoacetic acid, *F* Female partner, *ip-PPP* isopropylphenyl phenyl phosphate, *IVF* In vitro fertilization, *M* Male partner, *MeFOSAA* N-methylperfluorooctane sulfonamidoacetic acid, *PBB* polybrominated biphenyl, *PBDE* polybrominated diphenyl ether, *PFBA* perfluorobutanoic acid, *PFBS* perfluorobutanesulfonic acid, *PFDA* perfluorodecanoate, *PFDoA* perfluorododecanoic acid, *PFHpA* perfluoroheptanoic acid, *PFHpS* perfluoroheptanesulfonic acid, *PFHxA* perfluorohexanoic acid, *PFHxS* perfluorohexane sulfonate, *PFNA* perfluorononanoate, *PFOA* perfluorooctanoate, *PFOS* perfluorooctane sulfonate, *PFOSA* perfluorooctanesulfonamide, *PFTeDA* perfluorotetradecanoic acid, *PFTrDA* perfluorotridecanoic acid, *PFUnDA* perfluoroundecanoic acid, *SG* Specific gravity, *tb-PPP* tert-butylphenyl phenyl phosphate, *TTP* Time to pregnancy

Collectively, the existing data suggest that paternal preconception exposure to certain PFASs may influence specific pregnancy outcomes in a manner that differs from maternal exposure. However, additional research is needed given the limited evidence and the widespread nature of PFAS exposure. Further efforts to elucidate the mechanisms underlying the joint effects of both partners on multiple pregnancy endpoints are warranted, particularly in light of the distinct patterns observed for parental preconception PFAS exposure and infant sex.

### Flame Retardants

Brominated flame retardants (BFRs) were historically used in various consumer products to prevent the onset and spread of fire [85]. Since the 1970s, certain BFRs, such as polybrominated biphenyls (PBBs) and polybrominated diphenyl ethers (PBDEs), have been progressively phased out or banned in many countries due to health concerns [86]. However, these chemicals can leach out from products and persist in the environment, so contaminated air, dust, soil, and water continue to contribute to human exposure through inhalation, ingestion, or dermal contact [87]. In recent decades, organophosphate flame retardants (OPFRs) have increasingly been used as replacements for BFRs. OPFR metabolites, including diphenyl phosphate (DPHP) and bis(1,3-dichloro-2-propyl) phosphate (BDCIPP), have been detected in urine samples from most U.S. populations [22], including pregnant women [16]. However, OPFRs are also suspected endocrine disruptors, and emerging animal studies indicate potential reproductive and developmental toxicity, as reviewed elsewhere [88].

We identified seven epidemiological studies across four cohorts that examined the association between paternal exposure to brominated or organophosphate flame retardants and couples’ pregnancy outcomes (Table 3). Among 401 couples in the LIFE study [77], paternal serum PBDE-183 was suggestively associated with longer time-to-pregnancy (FOR = 0.86, 95% CI: 0.73, 1.01, per 1-SD increase in log-transformed concentrations). Notably, this analysis did not adjust for the female partner’s exposure, and no studies have yet examined OPFRs in relation to couples’ fecundity. In the EARTH study, paternal serum PBDEs were not associated with IVF treatment outcomes (i.e., fertilization, implantation, clinical pregnancy, live birth) [89]. However, paternal urinary BDCIPP, an OPFR metabolite, was inversely associated with the proportion of fertilized oocytes, with a marginal trend (*P* for trend = 0.06) [90]. Evidence on associations between paternal urinary DPHP and embryo quality comes from only two studies and is mixed: one study adjusting for the female partner’s DPHP reported no association [90], whereas another found an inverse association without such adjustment [39]. In the Michigan PBB Registry, where PBB was measured in blood samples collected from 1976 to 1978, paternal PBB exposure was associated with low birth weight, but not with gestational age, independent of maternal exposure [91]. Compared with the lowest tertile (< 3.0 part per billion), the multivariable-adjusted relative risk of low birth weight (< 3232 g) for the highest tertile of paternal serum PBB concentrations (9.0-1744.0 part per billion) was 2.06 (95% CI: 1.12, 3.79). In contrast, no association was observed between paternal preconception serum PBB concentrations and birth weight in the LIFE study, possibly because exposure levels were substantially lower [92]. Also, in the same study, higher paternal serum PBDE-183 was associated with reduced birth weight among female, but not male, infants [92]. Per 1-SD increase in log-transformed serum PBDE-183 concentrations in fathers, the adjusted mean changes in birth weight were − 92.1 g (95% CI: -173.4, -10.8) among 117 girls and 21.32 g (95% CI: -85.27, 127.91) among 113 boys. Only one publication from the LIFE study has examined the association between paternal PBB or PBDE exposure and offspring sex ratio, reporting null findings [93].

Overall, existing evidence suggests that paternal exposure to OPFRs may be associated with early reproductive outcomes among couples undergoing IVF treatment, and that paternal exposure to BFRs may adversely affect birth weight. As OPFRs are increasingly used as replacements for BFRs and emerging evidence links OPFR exposure to epigenetic changes in sperm DNA [94], further studies are needed to clarify how paternal OPFR exposure influences specific pregnancy endpoints in the general population.

## Conclusions

In this paper, we qualitatively reviewed existing epidemiological studies evaluating the relationships between paternal exposure to selected EDCs and couples’ pregnancy outcomes. Accumulating evidence suggests that paternal preconception exposure to certain phthalates may adversely affect pregnancy outcomes, whereas findings for paternal BPA and parabens remain largely null. Only a handful of studies have examined associations of paternal exposure to benzophenones, PFASs, and flame-retardant chemicals with specific pregnancy endpoints, yielding inconclusive results.

### Strengths and Limitations of Included Studies

**Fig. 1:**
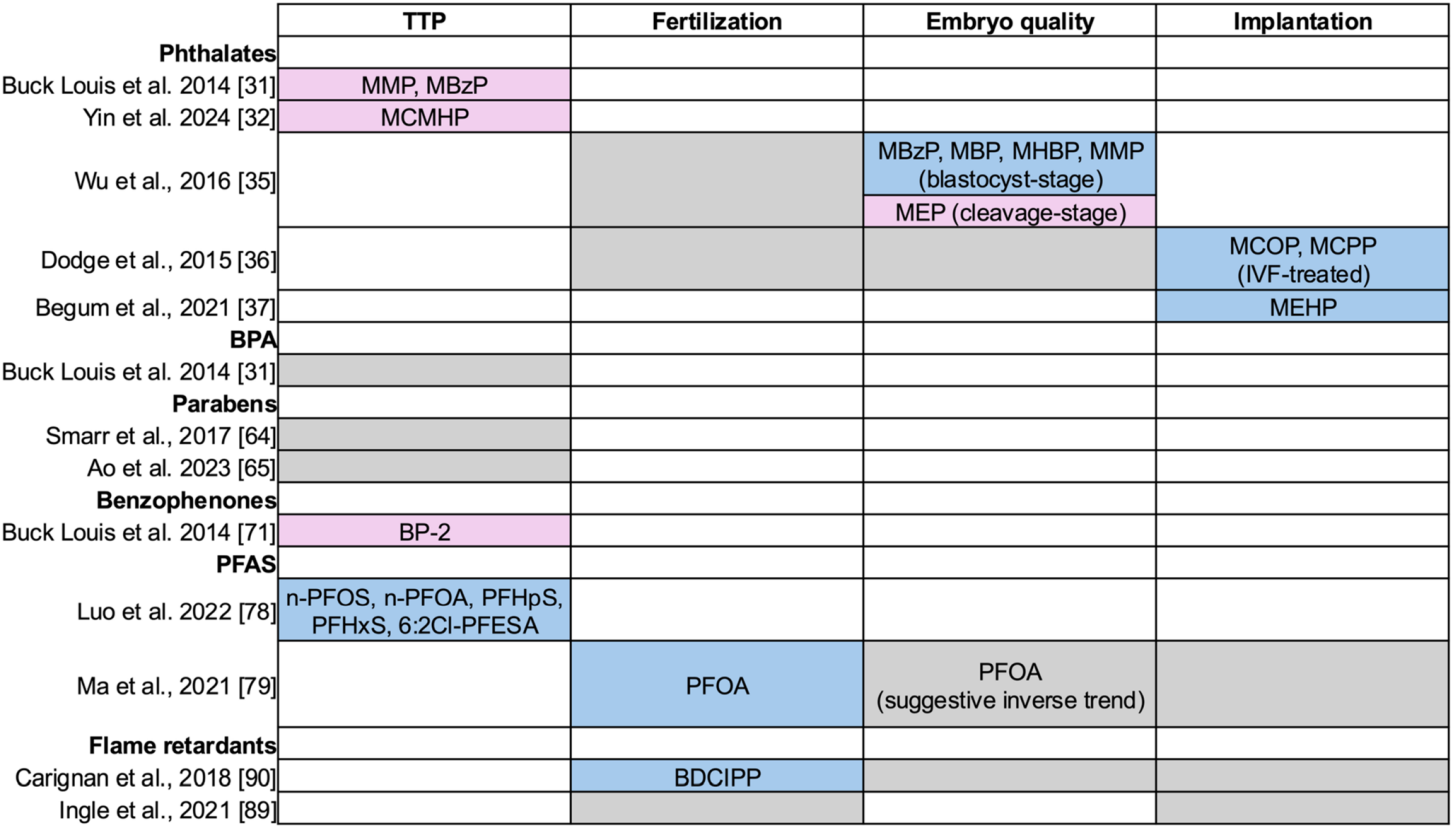
Associations between paternal EDC exposures and couples’ fecundity, fertilization, embryo quality, and implantation. Only prospective studies that adjusted for maternal exposure are included in the figure. Blue: inverse association. Pink: positive association. Grey: no significant association. Abbreviations: BDCIPP, bis(1,3-dichloro-2-propyl) phosphate; BP-2, benzophenone-2; BPA, bisphenol A; Cl: PFESA, chlorinated polyfluoroalkyl ether sulfonic acid; EDC, endocrine-disrupting chemical; IVF, in vitro fertilization; MBP, mono-n-butyl phthalate; MBzP, monobenzyl phthalate; MCMHP, mono(2-carboxymethylhexyl) phthalate; MCOP, monocarboxyisooctyl phthalate; MCPP, mono(3-carboxypropyl) phthalate; MEP, monoethyl phthalate; MEHP, mono(2-ethylhexyl) phthalate; MHBP, mono-3-hydroxybutyl phthalate; MMP, monomethyl phthalate; PFHpS, perfluoroheptanesulfonic acid; PFHxS, perfluorohexane sulfonate; PFOA, perfluorooctanoate; PFOS, perfluorooctane sulfonate; TTP, time to pregnancy

**Fig. 2:**
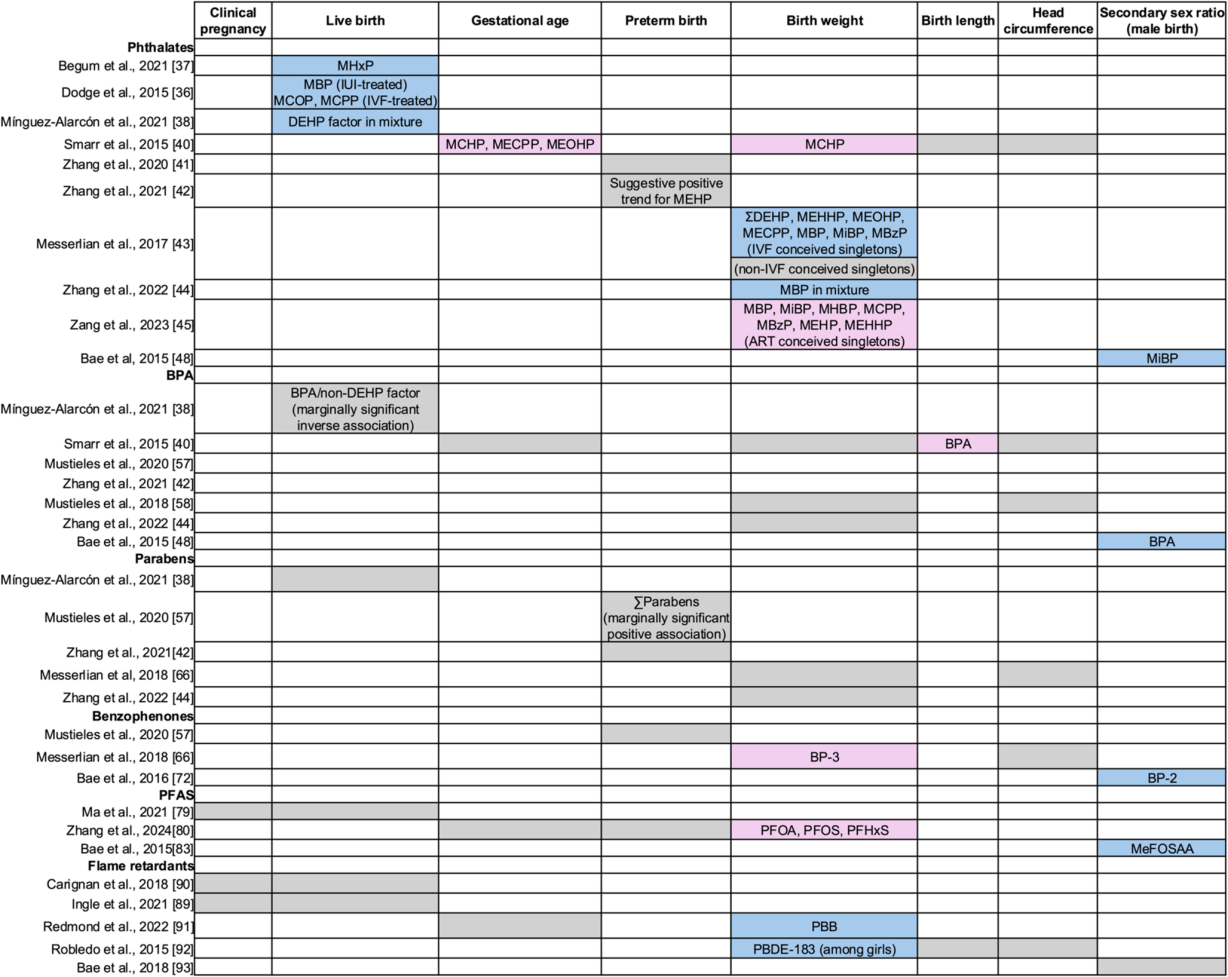
Associations between paternal EDC exposures and pregnancy and birth outcomes. Only prospective studies that adjusted for maternal exposure are included in the figure. Blue: inverse association. Pink: positive association. Grey: no significant association. Abbreviations: ART, assisted reproductive technology; BP-2, benzophenone-2; BP-3, benzophenone-3; BPA, bisphenol A; DEHP, di(2-ethylhexyl) phthalate; EDC, endocrine-disrupting chemical; IUI, intrauterine insemination; IVF, in vitro fertilization; MBP, mono-n-butyl phthalate; MBzP, monobenzyl phthalate; MCHP, mono-cyclohexyl phthalate; MCOP, monocarboxyisooctyl phthalate; MCPP, mono(3-carboxypropyl) phthalate; MECPP, mono(2-ethyl-5-carboxypentyl) phthalate; MeFOSAA, N-methylperfluorooctane sulfonamidoacetic acid; MEHHP, mono(2-ethyl-5-hydroxyhexyl) phthalate; MEHP, mono(2-ethylhexyl) phthalate; MEOHP, mono(2-ethyl-5-oxohexyl) phthalate; MHBP, mono-3-hydroxybutyl phthalate; MHxP, monohexyl phthalate; MiBP, mono-isobutyl phthalate; PBB, polybrominated biphenyl; PBDE, polybrominated diphenyl ether; PFHxS, perfluorohexane sulfonate; PFOA, perfluorooctanoate; PFOS, perfluorooctane sulfonate

Most of the existing evidence comes from two well-established cohort studies: the EARTH study, which recruited subfertile couples seeking fertility treatment, and the LIFE study, which enrolled couples who had discontinued contraception and were attempting to conceive. Their prospective, couple-based design and the assessment of exposure during the preconception period serve as one of the major strengths in examining the influence of paternal EDC exposure on couples’ pregnancy outcomes. However, concerns also arise about the generalizability of the findings, as potential cohort-specific selection bias may limit the extent to which the results can be applied to a broader population. In addition to studies from these two cohorts, some studies assessed paternal exposure only using measurements taken during pregnancy. Such a design is generally suboptimal: it may provide some information for chemicals with long biological half‑lives but is particularly unsuitable for short‑lived chemicals, as the most relevant exposures likely occur before conception. Moreover, some studies did not adequately account for maternal exposure, limiting the ability to identify the independent association of paternal exposure with pregnancy outcomes. As such, we focused on prospective studies that adjusted for maternal exposure and summarized these studies by specific pregnancy outcomes in Figs. 1 and 2. However, for most EDCs and specific endpoints, the evidence relies on only a few studies, precluding meaningful, systematic comparisons across chemicals and outcomes. Finally, although some EARTH analyses of birth outcomes used repeated measurements to characterize habitual preconception exposure, the majority of studies relied on a single measurement at enrollment, which may lead to exposure misclassification, particularly for EDCs with short half-lives such as phthalates and BPA. In addition, because some biospecimens were collected nearly two decades ago, they may not reflect temporal changes in exposure levels or allow adequate evaluation of emerging EDCs that have only appeared in more recent years.

### Future Prospectives

In addition to further investigating the EDCs covered in this review, emerging substances such as 1,2-dicarboxylic acid-diisononyl ester (DINCH) and di-(2-ethylhexyl) terephthalate (DEHTP) as traditional phthalate replacements, BPS and bisphenol F (BPF) as BPA replacements, or OPFRs as BFR replacements, are also critical targets given their increasing prevalence in recent years [16, 18, 21, 22]. Moreover, nontargeted analyses offer a promising strategy to identify novel chemical exposures that may be overlooked by traditional hypothesis-driven approaches focused only on targeted chemicals [95]. Emerging evidence indicates that mixture analyses can reveal complex joint effects of multiple paternal exposures on adverse pregnancy outcomes, which may be missed by single-chemical models [38, 42, 44]. Therefore, further research should also incorporate advanced mixture modeling approaches, alongside single-chemical analyses, to better characterize paternal EDC exposures and investigate their associations with couples’ pregnancy outcomes [96]. Although most studies on this topic have focused on non-Hispanic White populations, both exposure patterns and susceptibility to EDCs vary substantially by ethnicity, with women of color being particularly vulnerable to adverse effects of EDCs on pregnancy outcomes [97–99]. Therefore, additional research in more ethnically diverse populations is needed to confirm these associations and to provide further insight of the paternal perspective [100].

Overall, progress in this field will require large prospective studies with repeated preconception exposure assessments across diverse populations, integrated with mechanistic research such as the sperm epigenome, to confirm and elucidate how paternal exposure to specific EDCs or their mixtures affects pregnancy outcomes in couples.

## Data Availability

No datasets were generated or analysed during the current study.
